# Lanthanum phosphate/chitosan scaffolds enhance cytocompatibility and osteogenic efficiency via the Wnt/β-catenin pathway

**DOI:** 10.1186/s12951-018-0411-9

**Published:** 2018-11-29

**Authors:** Haoran Hu, Peipei Zhao, Jiayu Liu, Qinfei Ke, Changqing Zhang, Yaping Guo, Hao Ding

**Affiliations:** 1 Department of Orthopedic Surgery, Shanghai Jiao Tong University Affiliated Sixth People’s Hospital, Shanghai Jiao Tong University, Shanghai, 200233 China; 2 The Education Ministry Key Lab of Resource Chemistry and Shanghai Key Laboratory of Rare Earth Functional Materials, Shanghai Normal University, Shanghai, 200234 China

**Keywords:** Lanthanum, Nanoparticle, Bone defect, Osteogenesis, Wnt/β-catenin pathway

## Abstract

**Background:**

Fabrication of porous scaffolds with great biocompatibility and osteoinductivity to promote bone defect healing has attracted extensive attention.

**Methods:**

In a previous study, novel lanthanum phosphate (LaPO_4_)/chitosan (CS) scaffolds were prepared by distributing 40- to 60-nm LaPO_4_ nanoparticles throughout plate-like CS films.

**Results:**

Interconnected three dimensional (3D) macropores within the scaffolds increased the scaffold osteoconductivity, thereby promoting cell adhesion and bone tissue in-growth. The LaPO_4_/CS scaffolds showed no obvious toxicity and accelerated bone generation in a rat cranial defect model. Notably, the element La in the scaffolds was found to promote osteogenic differentiation of bone marrow mesenchymal stem cells (BMSCs) through the Wnt/β-catenin signalling pathway and induced high expression of the osteogenesis-related genes alkaline phosphatase, osteocalcin and Collagen I (Col-I). Moreover, the LaPO_4_/CS scaffolds enhanced bone regeneration and collagen fibre deposition in rat critical-sized calvarial defect sites.

**Conclusion:**

The novel LaPO_4_/CS scaffolds provide an admirable and promising platform for the repair of bone defects.

**Electronic supplementary material:**

The online version of this article (10.1186/s12951-018-0411-9) contains supplementary material, which is available to authorized users.

## Background

A substantial challenge remains in the treatment of bone defects caused by skeletal injury or bone disease [1–3]. Although autogenous bone grafts are recognized as the “gold standard”, their clinical application is restricted because of certain shortcomings, such as limited availability, additional pain, donor-position morbidity and infection risk [4]. Alternatively, various bone repair materials, including beta-tricalcium phosphate (β-TCP), hydroxyapatite (HA), bioactive glass (BG) and CS, have been recently developed [5, 6]. These biomaterials exhibit admirable biocompatibility and bioconductivity, but their limited osteoinductivity cannot meet the demands of patients, especially those with osteoporosis and metabolic disorders [7]. Consequently, developing a novel and osteoinductive platform for bone regenerations is a high priority.

The cell response and bone regeneration capacities of bone scaffolds can be improved by loading scaffolds with osteogenic growth factors/therapeutic drugs or doping with bioactive elements [8–10]. The introduction of bone morphogenetic protein 2, parathyroid hormone, vascular endothelial growth factor, bone forming peptide 1, or platelet derived growth factor in bone scaffolds effectively stimulates the osteogenic process of BMSCs and the formation of new bone tissues [11, 12]; however, the high concentrations of the released growth factors may cause side effects in the human body [13]. Another strategy is to incorporate bioactive elements in bone scaffolds to enhance cell proliferation, differentiation and bone regeneration [14–16]. Lanthanum (La), one of rare earth elements (REEs) found in the human body, participates in stem cell differentiation, tissue regeneration and metabolism [17]. La can delay vascular calcification for patients with hyperphosphataemic renal bone disease and shows positive phosphate binding effects in bone metabolism [18]. Osteoclast formation and function are attenuated by LaCl_3_ via down-regulation of rankl-induced Nf-κb and Nfatc1 activities [19]. In addition, lanthanum-containing nanoparticles have been reported as X-ray-mediated agents for tumour therapy [20]. Among the La-based bioceramics, LaPO_4_ nanoparticles have attracted special attention because of their intrinsic photoluminescence property, low toxicity and good drug delivery behaviour [21–23]. Up to now, LaPO_4_ nanoparticles have been widely used as imaging agents, drug carriers, and biomarkers [24, 25]. To the best of our knowledge, LaPO_4_-coordination scaffolds have rarely been reported as bone regeneration materials, and their role in osteogenesis remains unclear.

Human bone has a complex inorganic–organic porous architecture, in 3D connected macrochannels facilitate nutrition transference and vascular and bone tissue in-growth [26]. CS, a common polysaccharide that is non-toxic, naturally degrades and exhibits good biocompatibility, has been widely used for bone repair materials [16, 27, 28].

**Scheme 1:**
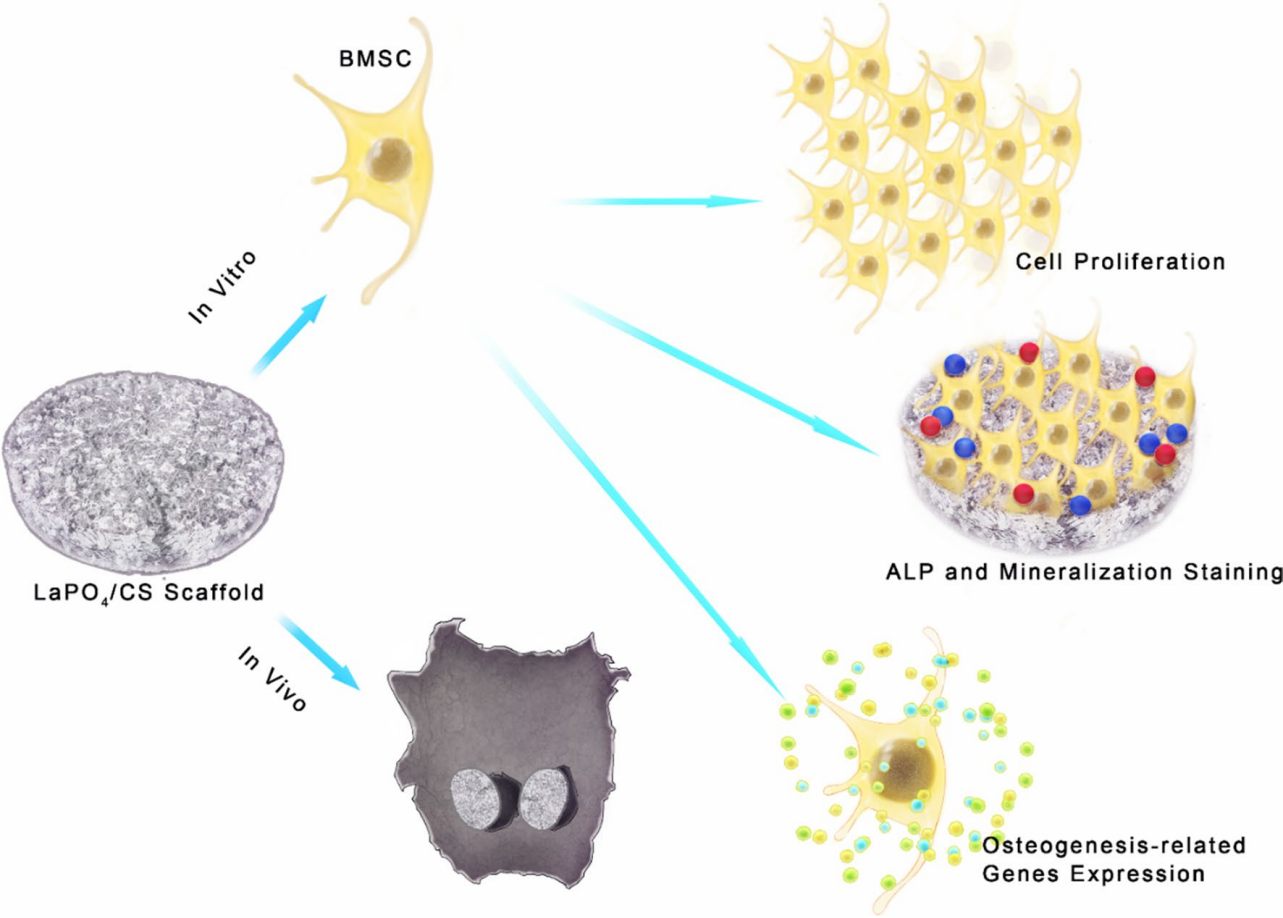
Lanthanum phosphate/chitosan scaffolds for bone defect therapy

Inspired by the architecture of natural bones, a novel and promising LaPO_4_/CS scaffold was constructed using a freeze-drying technique. This is the first report showing that LaPO_4_ nanoparticles in LaPO_4_/CS scaffolds rapidly promote the osteogenesis activity of BMSCs, and the underlying mechanism was attributed to Wnt/β-Catenin pathway activation (Scheme 1). A skull bone repair model in Sprague–Dawley (S–D) rats further demonstrated that the LaPO_4_/CS scaffolds significantly accelerate new bone regeneration compared with β-TCP/CS scaffolds. These exciting findings present a promising strategy for design and fabrication of novel REE-based biomaterials for skeletal treatments.

## Experimental section

### Preparation of LaPO_4_ nanoparticles

LaPO_4_ nanoparticles were fabricated by a chemical precipitation method. In brief, 2.9877 g La(NO_3_)_3_·6H_2_O and 0.9112 g (NH_4_)_2_HPO_4_ was separately dispersed in 100 mL deionized water. Then, the pH of an (NH_4_)_2_HPO_4_ solution was adjusted to 11–12 by adding NH_3_·H_2_O solution. The La(NO_3_)_3_ solution was dropwise dropped into the (NH_4_)_2_HPO_4_ solution under continuous agitation at room temperature (RT). After 2.5 h, the mixture was stirred further for 1 h at 90 °C and then stirred for 24 h at RT. The precipitates were washed with deionized water and alcohol until the products were neutral. Finally, LaPO_4_ nanoparticles were produced after drying at 60 °C for 24 h and calcination at 1000 °C for 3 h. Moreover, the β-TCP nanoparticles that served as the control group were prepared via a solid-state reaction [29, 30]. Calcium carbonate and ammonium phosphate (dibasic) mixtures at a molar ratio of 3:2 were calcined at 1000 °C for 3 h.

### Preparation of LaPO_4_/CS composite scaffold

Briefly, 4.0 g of CS powder was dissolved in 100 mL acetic acid solution (2.0 vol.%) to form a homogeneous CS solution. Then, 4.0 g of LaPO_4_ was added into the CS solution, and the solution was stirred for 2 h. The mixture was injected into wells in 24-well or 96-well plates. The samples were frozen at − 20 °C for 12 h and then freeze-dried in a freeze drier at − 60 °C for 48 h. The as-obtained precursor scaffolds were dipped in 0.1 mol/l sodium hydroxide solution for 24 h, followed by washing with deionized water for 6 days. β-TCP/CS scaffolds were prepared by the same method.

### Characterization

LaPO_4_ nanoparticles and LaPO_4_/CS scaffolds were coated with a thin gold layer, and then their morphologies were characterized by field-emission scanning electron microscopy (SEM; S-4800, Hitachi, Japan). The accelerating voltage was 5 kV and the working distance was kept at approximately 7 mm. The element compositions were determined by energy dispersive spectrometry (EDS; Quantax 400, Bruker) at an accelerating voltage of 20 kV. The microstructures of LaPO_4_ nanoparticles were characterized by high resolution transmission electron microscopy (HRTEM; JEM-2100, JEOL, Japan) and selected area electron diffraction (SAED) at 200kv. The phases of the samples were characterized by X-ray powder diffraction (XRD; D/Max-2200, Rigaku, Japan) using Cu Kα radiation at 40 kV with a scanning speed of 5º/min in the range of 2*θ* = 10°–70°. The functional groups in samples were detected by Fourier transform infrared spectroscopy (FTIR; Frontier, PerkinElmer, USA) in a wavenumber range of 4000–500 cm^−1^ with a resolution of 4 cm^−1^ at 100 ~ 230 V. A Malvern Zetasizer (Nano ZS90; Malvern Instruments Ltd. UK) was employed for dynamic light scattering (DLS) measurements after the LaPO_4_ particles were dispersed in ultrapure water. The organic and inorganic percentages of the scaffolds were calculated according to thermogravimetric analysis (TG; Perkin-Elmer) with a heating rate of 10 °C/min. The as-used purge gas was dry air with a flow rate of 50 mL/min and a heating rate of 10 °C/min.

### Cell isolation and culture

Rat bone marrow mesenchymal stem cells (BMSCs) were isolated from the femur of S–D rats using a Ficoll density gradient and then allowed to proliferate in minimum essential medium α (Gibco) with 10% foetal bovine serum (Gibco), penicillin (100 U/mL) and streptomycin (100 μg/mL) (Gibco) in an incubator with 5% CO_2_ at 37 °C. The cells used in this study were from passages 3–7.

### Cell morphology on scaffolds

To observe the attachment of cells on both LaPO_4_/CS and β-TCP/CS scaffolds, BMSCs were cultured on the scaffolds at a density of 2.0 × 10^4^ cells/mL in a 24-well plate. After 3 days of incubation, the cells were fixed with 2.5% glutaraldehyde, washed with phosphate buffer saline (PBS), dehydrated by ethanol, and freeze-dried at − 80 °C. Finally, SEM (S-4800, Hitachi, Japan) was utilized to observe the samples.

### Cell toxicity and proliferation on scaffolds

To estimate the cell toxicity and proliferation of the scaffold, on days 1, 3 and 7, Cell Counting Kit 8 (CCK-8; Dojindo, Japan) was adopted measure the cell viability. In brief, 1.0 × 10^4^ BMSCs were co-cultured with scaffolds in a 24-well plate for the given days. At each time point, 450 μL of culture medium with 50 μL (10%) of CCK-8 reagent was added to the samples. The cells were incubated for 2 h, and then, the absorbance at 450 nm was measured with a microplate reader (Bio-Rad, USA).

### Alkaline phosphatase (ALP) activity assay and staining

To carry out the ALP activity assay and staining, BMSCs were seeded on the LaPO_4_/CS and β-TCP/CS scaffolds for 7 and 14 days in a 24-well transwell plate at a primary density of 2.0 × 10^4^ cells/mL, with cells in the lower chamber and the scaffolds in the upper chamber. At each time point, the ALP activity was determined via the p-nitrophenyl-phosphate (pNPP) method, and ALP staining was conducted via the 5-bromo-4-chloro-3-indolyl phosphate/tetranitroblue tetrazolium chloride (BCIP/NBT) method according to the manufacturer’s instructions. For the ALP activity assay, the samples were washed twice with PBS and lysed with 0.1% Triton X-100, and then pNPP (Beyotime Biotechnology, China) was added for 60 min at 37 °C. Last, 1 M NaOH solution was added to quench the chromogenic reaction, and the absorbance at 405 nm of each sample was recorded. For ALP staining, a BCIP/NBT kit (Beyotime Biotechnology, China) was used to stain the samples, which were photographed with both a digital camera (Canon, Japan) and a microscope (Leica, Germany).

### Extracellular matrix (ECM) mineralization evaluation

Cells were cultured in a 24-well transwell plate. After 7 and 14 days of culture, the cells were fixed with 2.5% glutaraldehyde and washed twice with PBS. The samples were stained with 1 mM alizarin red (Cyagen, USA) for 10 min. Finally, images were acquired with both a digital camera and a microscope.

### Osteogenesis-related gene expression

Cells were seeded on the LaPO_4_/CS and β-TCP/CS scaffolds at a density of 2.0 × 10^4^ cells/mL in a 6-well plate for 7 and 14 days, and then, Trizol (Invitrogen) was used to extract messenger RNA (mRNA) from the cells, and Moloney Murine Leukemia Virus (M-MLV) Reverse Transcriptase (Takara) was used to synthesize the complementary DNA (cDNA). qPCRSuperMix (BioTNT), forward and reverse primers and cDNA were loaded onto a 384-well plate, and the reverse transcription-polymerase chain reaction (RT-PCR) procedure was performed using a Sequence Detection System (7900 HT, ABI). The primers used in this study are shown in Table 1, and GAPDH was used as the internal control gene.

**Table 1:** Real-time polymerase chain reaction primers used in this study

Gene	Accession number	Primers (up: forward; down: reverse)	Product size (bp)
GAPDH	NM_017008.4	GCAAGAGAGAGGCCCTCAG TGTGAGGGAGATGCTCAGTG	74
OCN	NM_013414.1	TCACTCTGCTGGCCCTGACT CCCTCCTGCTTGGACATGAA	100
ALP	NM_013059.1	CATCATCATGTTCCTGGGAG GACCTGAGCGTTGGTGTTGT	163
Col-I	NM_053304.1	GGCGAGTGCTGTCCTTTCTG CTTCCCCATCATCTCCGTTCTCTTCC	434

### Western blotting

The protein in rBMSCs from different groups was extracted with cell lysis buffer and proteinase inhibitor after 7 and 14 days of culture. A bicinchoninic acid Protein Assay Kit (Cell Signaling Technology) was used to quantify the protein concentrations. Each protein sample was loaded onto sodium dodecyl sulfate–polyacrylamide gel electrophoresis gels for electrophoresis (Millipore) and then transferred to polyvinylidene difluoride membranes. After blocking with Quickblock™ (Beyotime), the membrane was incubated with primary antibody targeting phosphorylated glycogen synthase kinase-3β (p-GSK3β), glycogen synthase kinase-3β (GSK3β), β-Catenin and GAPDH at 4 °C overnight and then with secondary antibodies at RT for 2 h.

### Procedures for Implantation of the Scaffolds into Animals

All animal surgical procedures were approved by the Animal Care and Experiment Committee of Shanghai Jiao Tong University Affiliated Sixth People’s Hospital. Twenty adult S–D rats (8 weeks old; weight 250 ± 25 g) were anaesthetized by intraperitoneal injection of chloral hydrate sodium (250 mg/kg). An incision with a sagittal length of 1.5–2.0 cm was made on the scalp, and then, two full-thickness 5-mm-diameter defects were made on both sides of the skull with an electric trephine drill. The scaffolds implanted were 5 mm in diameter and 2 mm in thickness and randomly implanted into each cranial defect. The rats were intraperitoneally injected with fluorochromes under anaesthesia as follows: 25 mg/kg tetracycline (Sigma, USA) on week 3, 30 mg/kg alizarin red (Sigma, USA) on week 6 and 20 mg/kg calcein (Sigma, USA) on week 9. On week 12, all rats were sacrificed by intraperitoneal injection with an overdose of hydrate sodium, and the calvarias were obtained.

### Micro-CT and histomorphometric analyses

The skull specimens were scanned using micro-computerized tomography (micro-CT, Skyscan, Belgium). Scanning was performed with a 100 kV/100 μA X-ray source with an isotropic voxel size of 18 μm. The images were used to reconstruct tomograms with a 3D Creator software (Skyscan Software). Bone mineral density (BMD) and the ratio of bone volume/tissue volume (BV/TV) were measured with CTAn (Skyscan Software) based on the reconstructed micro-CT images.

### Histological analysis

For histological analysis, the specimens were dehydrated in a graded alcohol series for 3 days and then embedded in methyl methacrylate without decalcification. The non-decalcified samples were cut with a diamond saw (SP1600, Leica) and ground to approximately 150 μm in thickness. After fluorescence labelling, the specimens were evaluated with a confocal laser scanning microscope (Leica). After Van Gieson’s (VG) staining, the specimens were observed with an optical microscope (Leica).

### Statistical analysis

Statistical analyses were performed using SPSS 20.0 (SPSS Inc., Chicago, IL, USA). All the data were expressed as mean ± standard deviation and analysed by one-way analysis of variance with Bonferroni’s post hoc test. Statistical significance was indicated when p < 0.05.

## Results

### Morphologies and Structures of LaPO_4_/CS Scaffolds

**Fig. 1:**
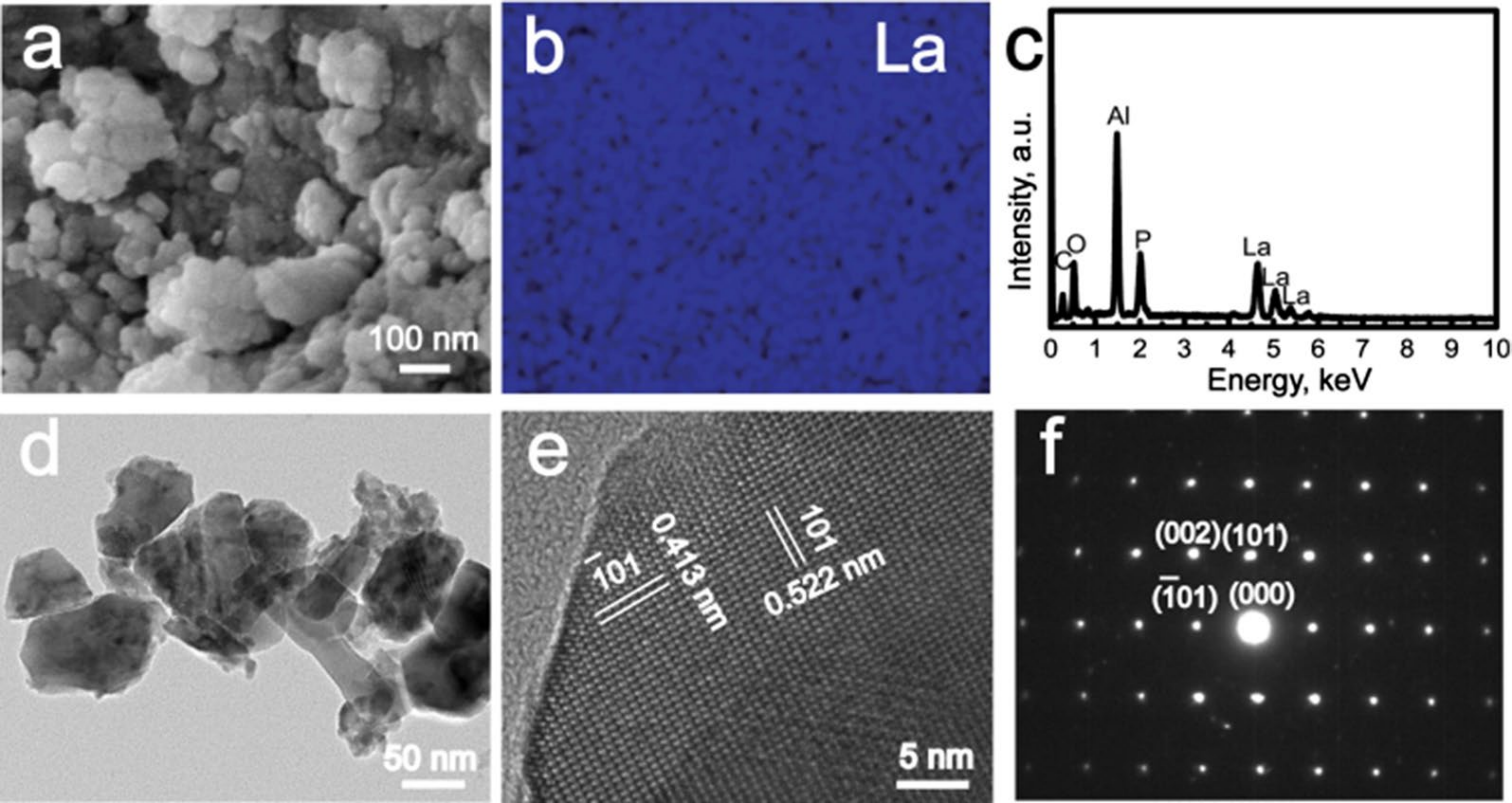
Characterization of LaPO_4_ nanoparticles: **a** SEM image; **b** image showing La distribution; **c** EDS spectrum; **d** TEM image; **e** HRTEM image; **f** SAED pattern

Inspired by the hierarchical architecture of bone, LaPO_4_/CS scaffolds were constructed according to the following steps: (i) coprecipitation preparation of LaPO_4_ nanoparticles; and (ii) freeze–drying synthesis of the LaPO_4_/CS scaffolds. SEM and TEM images showed that the as-obtained LaPO_4_ nanoparticles exhibited irregular shapes with sizes of approximately 40–60 nm (Fig. 1a, d). The nanostructure of LaPO_4_ can increase the surface energy, easily resulting in the agglomeration of these nanoparticles (Fig. 1a). The hydrodynamic sizes of LaPO_4_ agglomerates were mainly distributed at around 500 nm, as detected by a Malvern Zetasizer Nano ZS90 instrument (Additional file 1: Figure S1). As shown in the EDS spectrum (Fig. 1c), the elements La, P and O were present in the LaPO_4_ nanoparticles, and Al was present in the aluminium foil. La was uniformly dispersed within the LaPO_4_ nanoparticles (Fig. 1b). A representative HRTEM image taken from an individual LaPO_4_ nanoparticle showed a lattice spacing of 4.138 Å and 5.227 Å, which can be indexed to the (${\bar{\text{1}}}0 1$) and (101) crystal planes of LaPO_4_, respectively (Fig. 1e). The SAED pattern the LaPO_4_ nanoparticles exhibited bright spots, indicating a monocrystal structure with a [$0 1 {\bar{\text{1}}}$] zone axis.

**Fig. 2:**
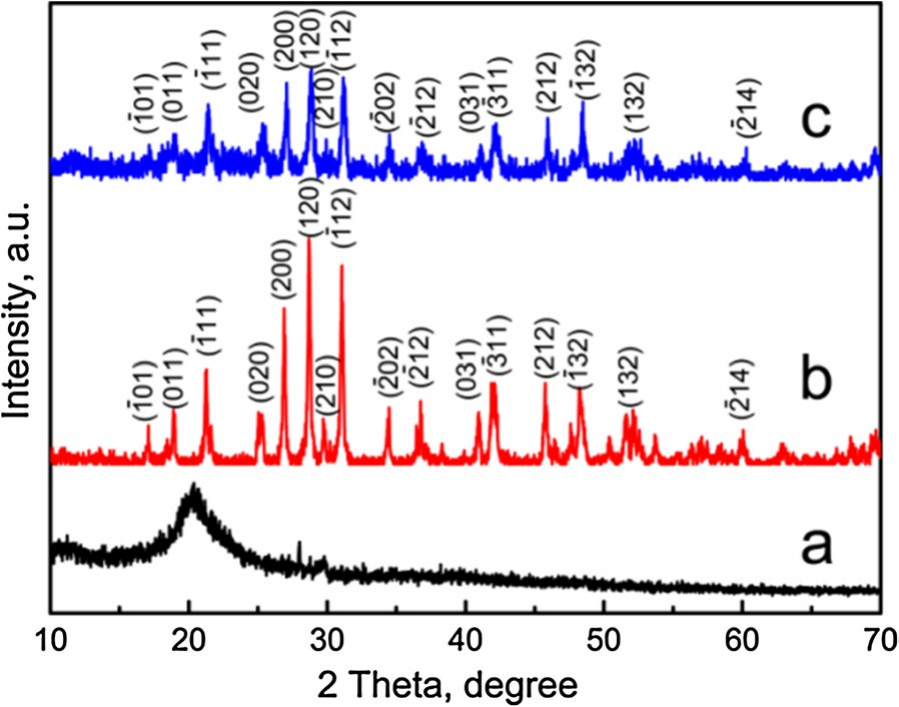
XRD patterns of samples: **a** CS; **b** LaPO_4_ particles; **c** LaPO_4_/CS composite scaffold

The phases of the LaPO_4_/CS scaffolds were investigated by XRD patterns using pure CS and LaPO_4_ as control groups (Fig. 2). The characteristic peaks of LaPO_4_ nanoparticles indicated a monoclinic phase with a space group of *P*21/*n*(14) (JCPDS 16-0382) [31]. As we know, the structure units of CS include β-(1,4)-2-acetamido-2-β-d-glucose and β-(1,4)-2-amido-2-β-d-glucose units [32]. The diffraction peaks at approximately 20° and 28° revealed that the pure CS exhibited semi-crystalline features (Fig. 2a). Compared with the pure CS and LaPO_4_, only characteristic peaks of LaPO_4_ were detected in the LaPO_4_/CS scaffolds due to the lower crystallinity of CS relative to LaPO_4_ (Fig. 2).

**Fig. 3:**
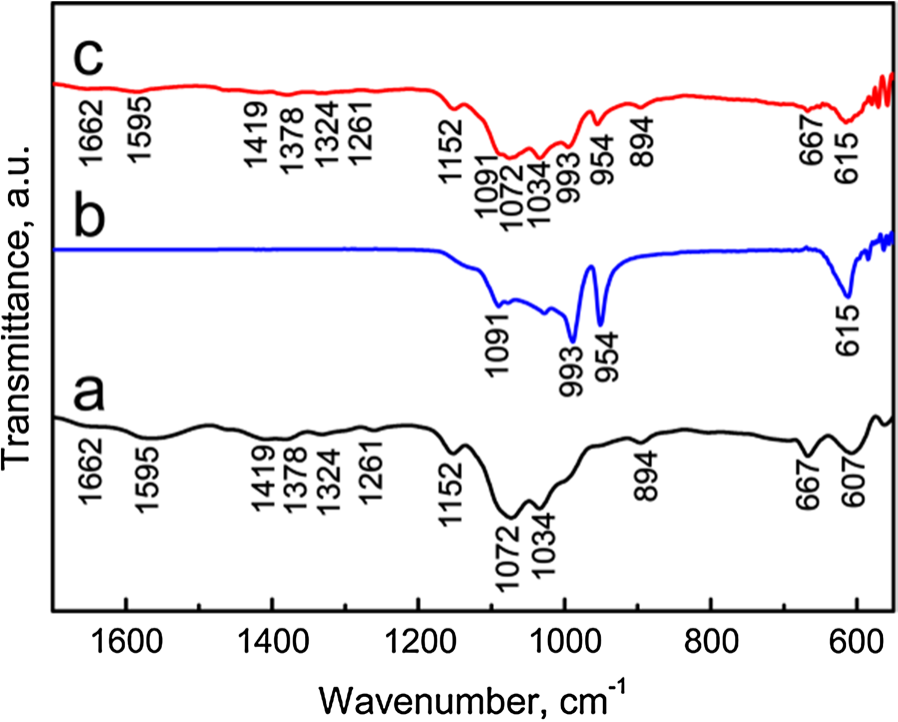
FTIR spectra of samples: **a** CS; **b** LaPO_4_ particles; **c** LaPO_4_/CS composite scaffold

The functional groups of the LaPO_4_/CS scaffolds were demonstrated by FTIR spectra using pure CS powders and LaPO_4_ as control groups. The LaPO_4_/CS scaffolds possessed the characteristic bands of both LaPO_4_ and CS (Fig. 3). For the LaPO_4_/CS scaffolds and CS powders (Fig. 3a, c), the bands at 1662, 1595 and 894 cm^−1^ corresponded to the amide-I vibration, N–H deformation vibration and N–H wagging vibration in amino groups, respectively [33]. The C-O stretching vibration bands were located at 1072/1034 cm^−1^, and the bridge oxygen stretching vibration bands were located at 1152 cm^−1^ [34]. The characteristic bands of PO_4_^3-^ groups were observed in both the LaPO_4_/CS scaffolds and LaPO_4_ nanoparticles. The P-O antisymmetric stretching vibration (*v*_3_) band was located at 993 and 954 cm^−1^, and the antisymmetric deformation vibration (*v*_4_) band was located at 615 cm^−1^ [35, 36].

**Fig. 4:**
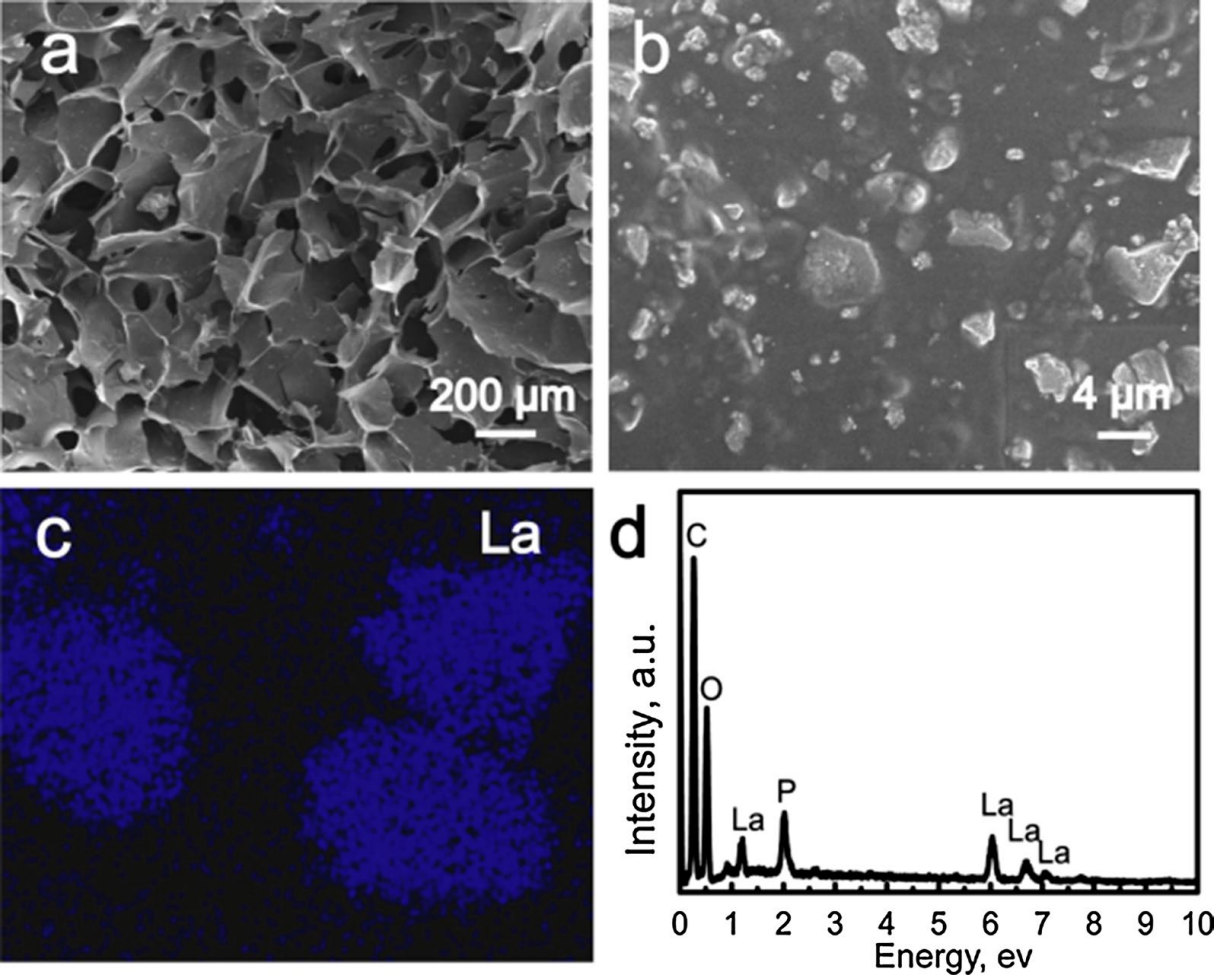
Characterization of LaPO_4_/CS scaffolds: **a**, **b** SEM images; **c** surface scan map of La; **d** EDS spectrum

The morphologies of LaPO_4_/CS scaffolds were characterized by SEM, as shown in Fig. 4. The LaPO_4_/CS scaffolds possessed 3D interconnected macrochannels with pore sizes of approximately 200 µm (Fig. 4a). These interlinked macropores, which were formed due to volatilization of ice crystals during the freeze-drying procedure, can promote body fluid exchange and bone tissue in-growth [26]. The CS films were connected together, and the macropores were present among the films. Moreover, the LaPO_4_ nanoparticles were distributed within or on the CS films, and they presented a granule-stacking structure because of their small particle sizes (Fig. 4b). A La surface scan map of La further demonstrated the presence of LaPO_4_ nanoparticles within the scaffolds (Fig. 4c). The EDS spectrum indicated that the LaPO_4_/CS composite scaffolds were composed of the elements La, P, O and C (Fig. 4d). The elements La and P were ascribed to the LaPO_4_ nanoparticles, and C was ascribed to the CS. The TG curves indicated that the volatilization of adsorbed water took place at 40–100 °C, while the decomposition of CS occurred at 100–550 °C (Additional file 1: Figure S2). The percentages of different components in the scaffolds could be calculated according to the TG curves. The percentages of adsorption water, LaPO_4_ and CS in the LaPO_4_/CS scaffolds were approximately 9%, 47% and 44%, respectively (Additional file 1: Figure S2a). The percentages of adsorption water, LaPO_4_ and CS in the LaPO_4_/CS scaffolds were approximately 7%, 47% and 46%, respectively (Additional file 1: Figure S2b).

### In Vitro Cytocompatibility and Osteogenesis Ability of LaPO_4_/CS Scaffolds

**Fig. 5:**
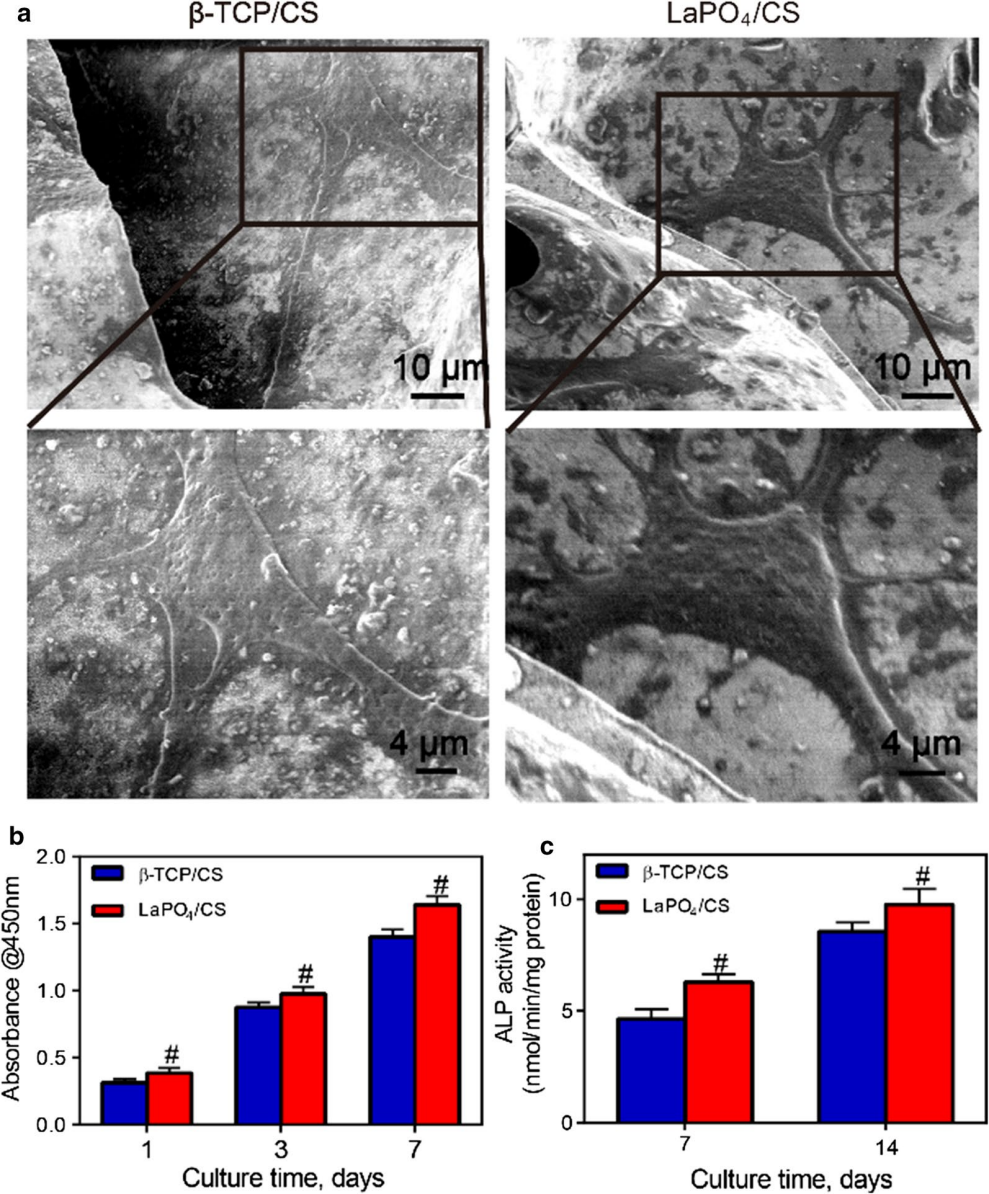
**a** SEM images of the LaPO_4_/CS and β-TCP/CS scaffolds after 3 days of incubation with rBMSCs. **b** CCK-8 results on days 1, 3 and 7; **c** ALP activity results on days 7 and 14 (#: a significant difference compared with the β-TCP/CS group (*p *<* 0.05*))

The cytocompatibility and osteogenesis ability of the LaPO_4_/CS scaffolds were assessed using rBMSCs as the cell model and β-TCP/CS scaffolds as the control group. SEM images indicated that the rBMSCs attached tightly to the films of both the LaPO_4_/CS scaffolds and β-TCP/CS scaffolds after 3 days of culture (Fig. 5a). Long cell pseudopodia spread well and stretched gradually into the scaffold interior through the interconnected macropore channels. The cells on both the LaPO_4_/CS scaffolds and β-TCP/CS scaffolds continuously proliferated from day 1 to day 7, as shown by the CCK-8 results (Fig. 5b). Notably, the cell number on the LaPO_4_/CS scaffolds was significantly greater than that on the β-TCP/CS scaffolds (^#^*p *< 0.05, Fig. 5b) after culture for 1, 3 or 7 days, suggesting that the LaPO_4_/CS scaffolds remarkably supported the proliferation of rBMSCs compared with the β-TCP/CS scaffolds.

**Fig. 6:**
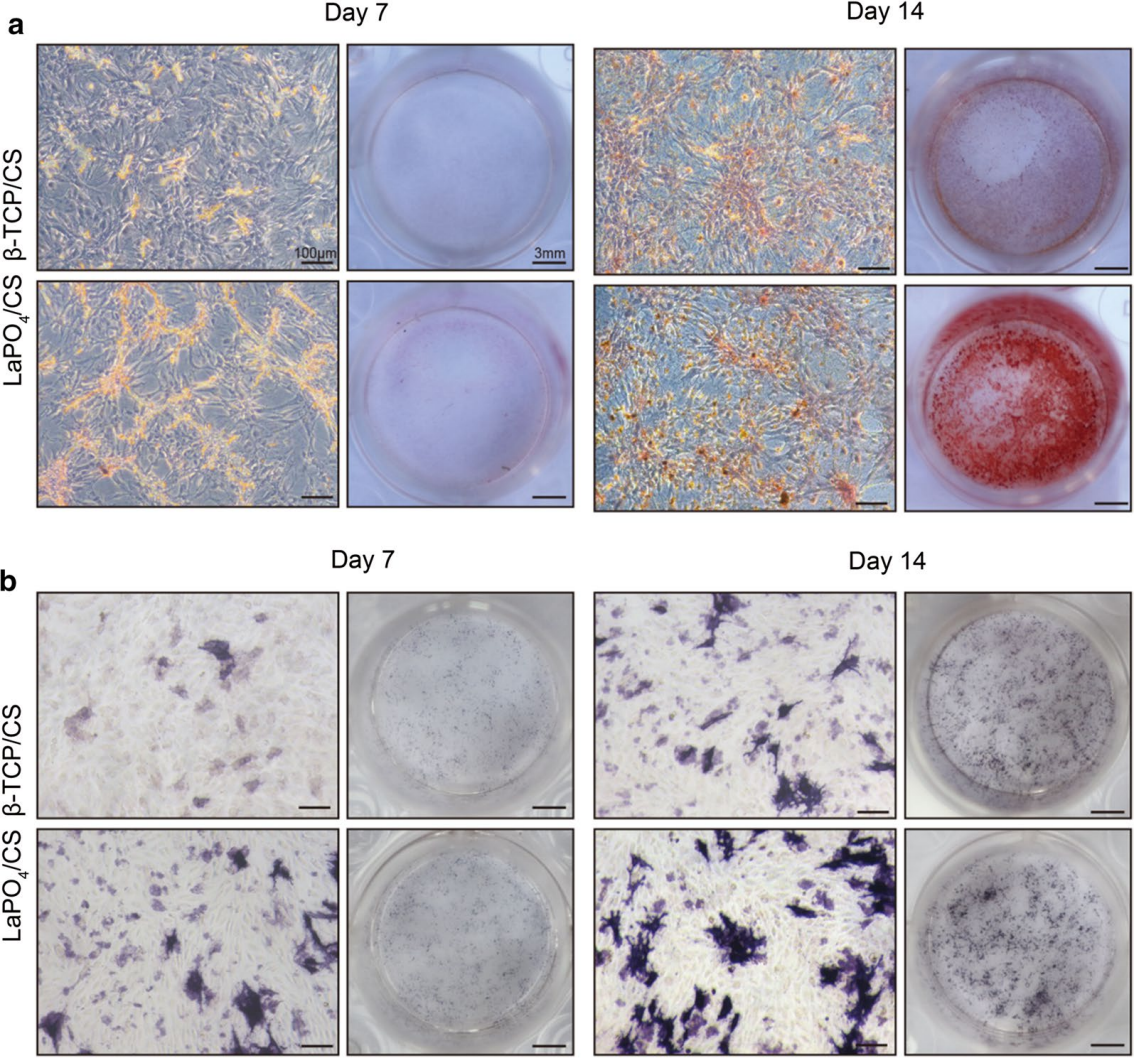
**a** Alizarin red staining and **b** ALP staining results shown via macroscopic and microscopic observation after cell culture for 7 and 14 days

The osteogenesis ability of the LaPO_4_/CS scaffolds was determined by ALP activity, ALP staining and mineralization evaluation assays. ALP activity is an early osteogenic differentiation marker secreted by stem cells. The ALP activity in the LaPO_4_/CS group was significantly higher than that in the β-TCP/CS group (Fig. 5c, ^#^*p *< 0.05). ALP staining images further demonstrated that the cells co-cultured with the LaPO_4_/CS scaffolds produced more ALP than those with cultured with the β-TCP/CS scaffolds at days 7 and 14 (Fig. 6b). Meanwhile, macroscopic and microscopic alizarin red staining images revealed that the rBMSCs in the LaPO_4_/CS group generated more ECM mineralization than those in the β-TCP/CS group at days 7 and 14 (Fig. 6a). Taken together, the in vitro cell tests suggest that both the LaPO_4_/CS and β-TCP/CS scaffolds present excellent cytocompatibility, and the LaPO_4_/CS scaffolds exhibited better osteogenic effects than the β-TCP/CS scaffolds.

**Fig. 7:**
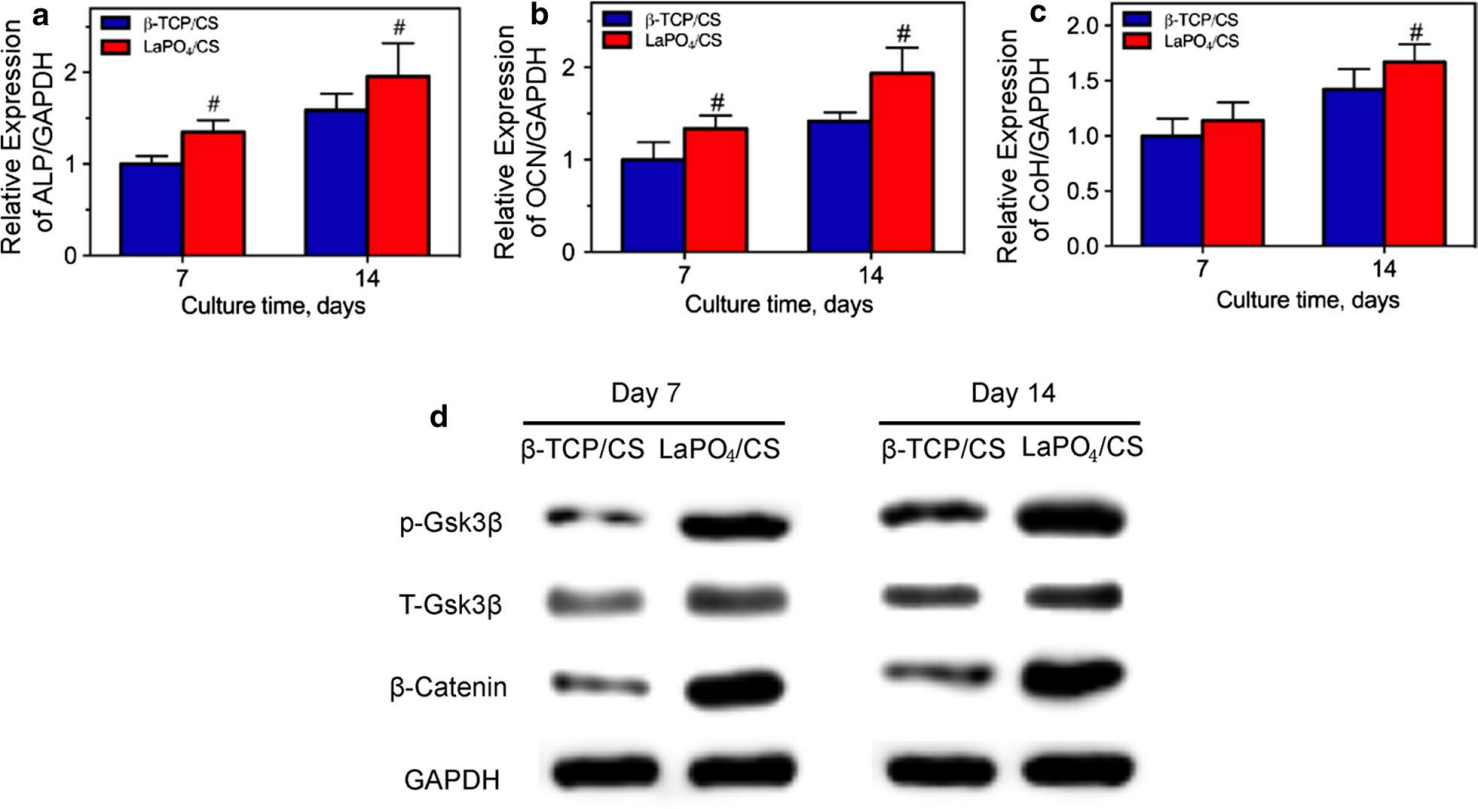
mRNA expression of osteogenic-related genes in rBMSCs, ALP (**a**), OCN (**b**) and Col-I (**c**), on days 7 and 14 (^*#*^a significant difference compared with β-TCP/CS (*p *<* 0.05*)). **d** p-Gsk3β, Gsk3β, β-Catenin and GAPDH expression measured via western blotting at days 7 and 14. GAPDH was used as the internal reference

To further investigate the underlying osteogenesis mechanism of the LaPO_4_/CS scaffolds, the expression of osteogenesis-related genes in the BMSCs co-cultured with the LaPO_4_/CS and β-TCP/CS scaffolds was evaluated by RT-PCR and western blot analysis (Fig. 7). After culture for 7 or 14 days, the expression levels of ALP and osteocalcin (OCN) in the LaPO_4_/CS group were significantly increased compared with those in the β-TCP/CS group (Fig. 7a, b). Although the expression level of Col-I showed no significant difference between the LaPO_4_/CS and β-TCP/CS group at day 7, the Col-I expression level in the former was much greater than that in the latter at day 14 (Fig. 7c). Moreover, western blotting results revealed that the LaPO_4_/CS scaffolds obviously enhanced the expression of phosphorylated Gsk3β and β-Catenin compared with the β-TCP/CS scaffolds (Fig. 7d). Gsk3β and β-Catenin are key proteins in the Wnt/β-catenin pathway. Therefore, it can be inferred that the LaPO_4_/CS scaffolds enhance osteogenesis activity by activating the Wnt/β-catenin signalling way.

### Bone regeneration ability in vivo

**Fig. 8:**
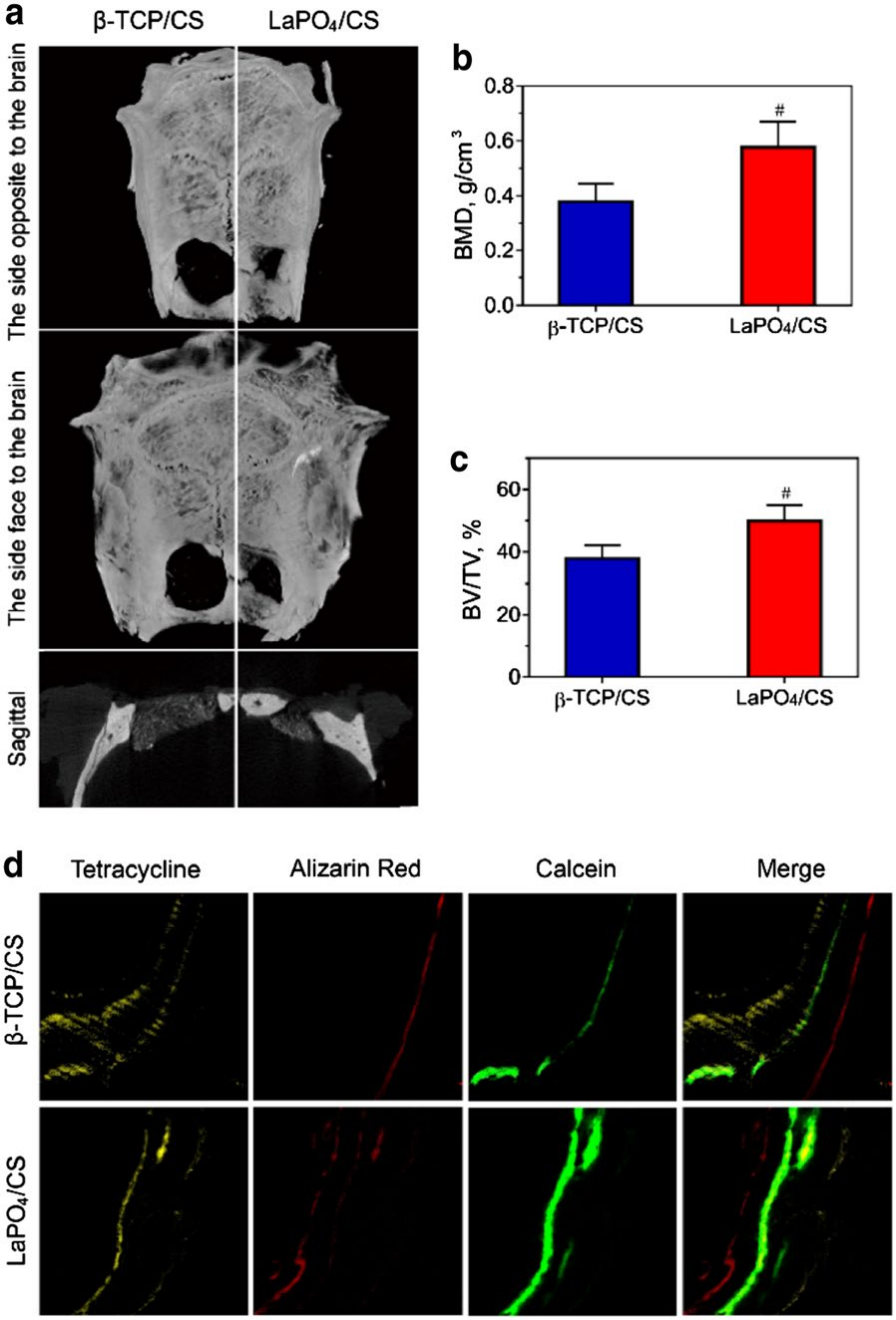
**a** Micro-CT results of 3D reconstructed and 2D sagittal images and the BMD (**b**) and BV/TV (**c**) results (^*#*^a significant difference compared with β-TCP/CS, *p *< *0.05*). **d** New bone formation and mineralization determined by fluorochrome-labelling analysis. Row 1 (yellow) shows tetracycline at week 2, row 2 (red) shows alizarin red at week 4, row 3 (green) shows calcein at week 6, and row 4 shows merged images of the three fluorochromes for the same group

The in vivo bone formation capacity of the LaPO_4_/CS scaffolds was evaluated using critical-sized calvarial bone defect models. 3D reconstructed micro-CT images indicated that the LaPO_4_/CS scaffolds had a better ability to promote new bone formation in the cranial defects than the β-TCP/CS scaffolds after 12 weeks of implantation (Fig. 8a). Simultaneously, the calculated BMD and BV/TV values confirmed the above conclusion. The LaPO_4_/CS group showed a higher BMD (0.577 ± 0.053) than the β-TCP/CS group (0.379 ± 0.037) (Fig. 8b, *p *< 0.05). The BV/TV ratio in the LaPO_4_/CS scaffold group was also increased up to 49.87 ± 2.91% compared with the β-TCP/CS group (37.85 ± 2.44%) (Fig. 8c, ^*#*^*p *< *0.05*).

**Fig. 9:**
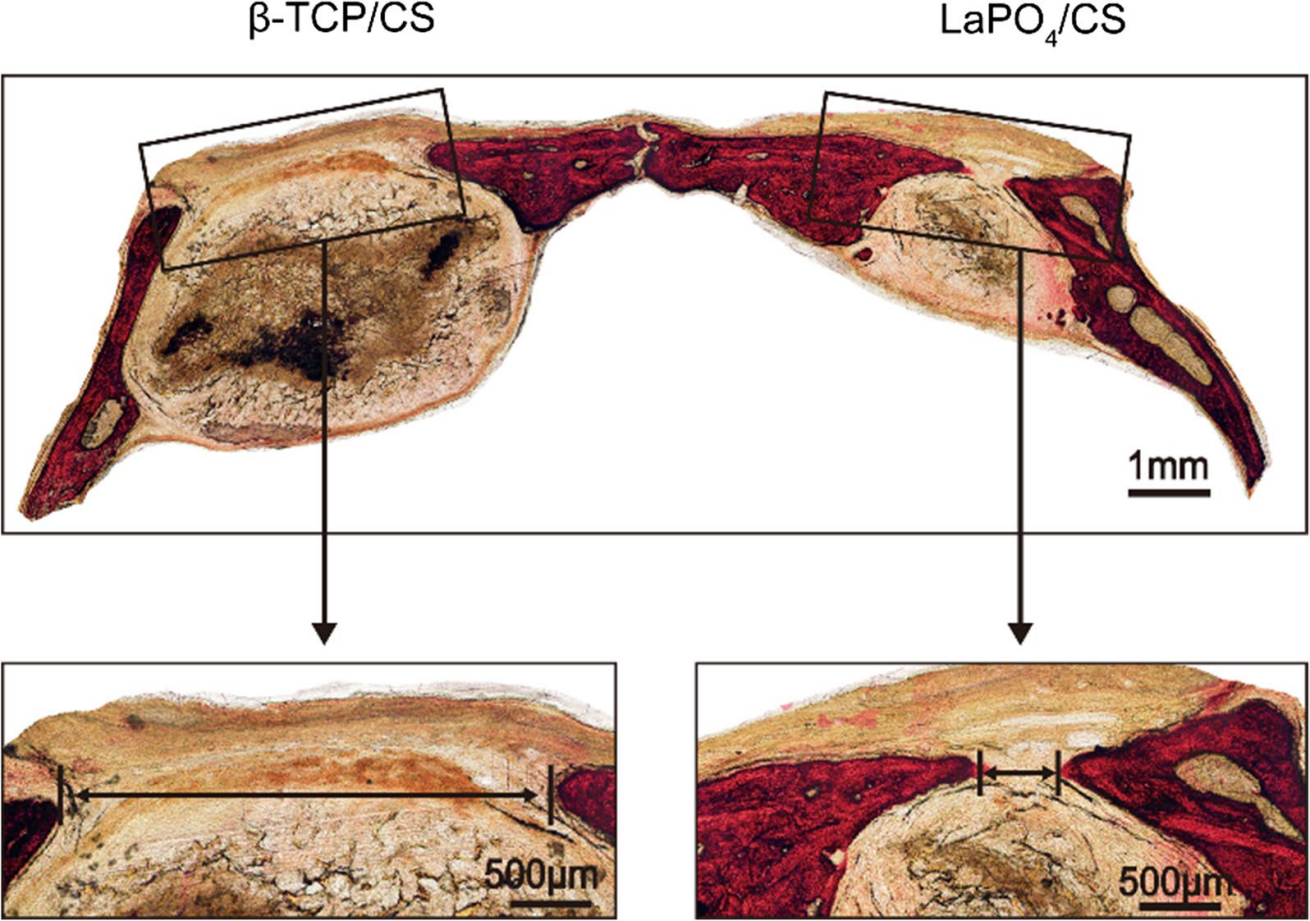
Transmitted light images of Van Gieson’s picrofuchsin-stained sections of rat calvarial defects with implanted scaffolds at 12 weeks post-implantation. New bone is shown in red, and the scaffold is shown in black

The newly formed bone was characterized at different weeks post-operation via histological analysis of triple fluorescence labelling, specifically tetracycline (yellow) at week 3, calcein (green) at week 6 and alizarin red (red) at week 6 (Fig. 8d). Stronger density and a more widely spread area of fluorescence were detected in the LaPO_4_/CS group than in the β-TCP/CS group. Moreover, VG staining (Fig. 9) suggested more collagen fibres were present surrounding the sites of the LaPO_4_/CS scaffolds than surrounding the β-TCP/CS scaffolds, indicating that the LaPO_4_/CS scaffolds exhibited better potential for collagenous matrix formation. Taken together, the micro-CT reconstruction, fluorescence labelling and VG staining results demonstrated that the LaPO_4_/CS scaffolds could better enhance both in vivo new bone formation and collagen formation than the β-TCP/CS scaffolds.

## Discussion

Developments in nanotechnology, molecular biology and nanomedicine have facilitated the fabrication of various biomedical materials [37, 38]. To date, a large number of bone repair materials have been developed for treating bone defects; however, their low bone-forming ability still cannot meet the demands of patients, especially those with osteoporosis and metabolic disorders [6]. Notably, REEs play a pivotal role in regulating the function and performance of bone tissues. Hence, here, novel LaPO_4_/CS scaffolds were developed for the first time for use as bone repair materials.

Excellent osteoconductivity and biocompatibility are prerequisites for bone tissue engineering materials. It has been reported that CS has biological and chemical similarities to natural tissues, which could promote the proliferation of mesenchymal cells such as BMSCs and stimulate its differentiation [39, 40]. Combination of natural chitin-calcium-carbonate-protein complexes can result in hardening of the CS scaffold [41]. The 3D CS-based scaffolds were reported to provide a proper space for new bone tissue regeneration with optimal size of 200-600 µm to facilitate osteoconduction and bone growth [42]. During the freeze-drying process, a 3D interconnected macroporous structure with pore sizes of approximately 200 μm was produced by using ice crystals as templates (Fig. 4). The large macropores not only provided enough spaces for cell adhesion and pseudopodium migration (Fig. 5a) but also supported the in-growth of bone tissues and cells (Figs. 7 and 8).

Moreover, the LaPO_4_/CS scaffolds exhibited excellent biocompatibility, and the chemical components in the scaffolds were non-toxic. On one hand, BMSCs cultured on the scaffolds proliferated continuously from 1 to 7 days (Fig. 5b); on the other hand, no rejection reaction was observed after implantation of the scaffolds into calvarial bone defect sites (Fig. 9). The above in vitro and in vivo results suggest that LaPO_4_**/**CS scaffolds offer great osteoconductivity, cytocompatibility and histocompatibility.

The osteoinductivity of bone scaffolds plays a key role in inducing in vivo new bone formation. To enhance osteogenesis activity, various bioactive elements such as Li, Mg and Sr, are widely incorporated into bone scaffolds [14–16]. For example, Mg^2+^ ions released from implants induce overexpression of calcitonin receptor-like receptor and receptor activity-modifying protein, and thus promote new bone growth [15]. For strontium hydroxyapatite [SrHAP, Ca_10−*x*_Sr_*x*_(PO_4_)_6_(OH)_2_], the synergetic effect between Ca^2+^ and Sr^2+^ ions remarkably increases osteogenic-related gene expression levels and promotes ECM mineralization [16]. La has been reported to penetrates all intercellular spaces and the newly forming bone matrix to enhance the mineral deposition [43], showing its osteoblast potential in tissue engineering. Recent years, La has also been tested to improve proliferation, osteogenic differentiation and mineralization in vitro [44] and stimulates bone formation in a rat model in vivo [45]. In this work, relatively integrated in vivo and in vitro experiments were carried out to obtain a comprehensive understanding of the effects of La^3+^ on the osteogenesis process. Compared with the β-TCP/CS scaffolds, the LaPO_4_/CS scaffolds presented a better cell proliferation performance (Fig. 5b), indicating positive effects of La on BMSCs. Moreover, the LaPO_4_**/**CS scaffolds induced higher ALP activity and ECM mineralization than the β-TCP/CS scaffolds, as demonstrated by the ALP activity assay, ALP staining and alizarin red staining (Figs. 5c and 6), which accelerates new bone formation and mineralization.

To determine the mechanism of LaPO4/CS scaffolds in osteogenesis, RT-PCR analysis and western blotting were employed to measure the expression levels of osteogenic-related genes and proteins. The RT-PCR analysis (Fig. 7a–c) revealed that the expression of ALP, OCN and Col-I were up-regulated by the LaPO_4_**/**CS scaffolds. ALP is one of the key enzymes in BMSC differentiation and osteogenesis [46], and higher ALP expression indicates an enhancement of bone formation in vitro. OCN and Col-I are components of mature bone, and their presence indicates early ECM mineralization. Meanwhile, western blotting was used to assess the cellular signalling processes in BMSCs and verified that LaPO4/CS scaffolds up-regulated the expression of Wnt/β-catenin pathway components (Fig. 7d).

The Wnt/β-catenin pathway is an important pathway that regulates osteogenic differentiation activities [47]. The pathway plays a central role in the bone regeneration and is an important regulator of bone formation in the process of osteoblastic differentiation. It also stimulates its downstream pathways that result in affecting several osteogenic processes including not only osteoblast attachment and differentiation but also cell maturation and apoptosis [48]. Moreover, the pathway has also been reported to promote both mineral deposition in ECM and the repair of tissue defect [49, 50]. The proteins of β-Catenin and Gsk3β are key proteins in the canonical Wnt/β-catenin pathway. The western blotting results suggested that a higher level of GSK3β phosphorylation and increased expression of β-Catenin were induced by LaPO_4_/CS scaffolds, and this enhancement suggested up-regulation of the Wnt/β-catenin pathway, which would improve cell proliferation and osteogenic differentiation.

In addition, an in vivo study with rat critical-sized calvarial defect sites was conducted to confirm the biofunction of LaPO_4_/CS scaffolds in bone repair. 3D reconstruction of micro-CT images (Fig. 8a) allowed visualization of the effects of LaPO_4_/CS scaffolds, and the results were verified by quantitative evaluation of BMD and BV/TV (Fig. 8b, c). Meanwhile, triple fluorochrome and VG staining also showed more bone and collagenous matrix formation at the defect site (Figs. 8d and 9). In summary, LaPO_4_/CS scaffolds exhibit promising potential for bone regeneration both in vitro and in vivo.

## Conclusion

In the present study, LaPO_4_/CS scaffolds were prepared for bone defect healing. LaPO_4_ nanoparticles with sizes of 40–60 nm were evenly distributed throughout the surface of the scaffolds. Interconnected 3D macropores were formed due to the connection of the plate-like films, which increased the osteoconductivity of the LaPO_4_/CS scaffolds for cell adhesion and bone tissue in-growth. The LaPO_4_/CS scaffolds showed no obvious toxicity or effects on cell morphology, and they accelerated bone generation in a rat cranial defect model. More importantly, controlled release of La^3+^ from the scaffolds was found to promote osteogenic differentiation of BMSCs through the Wnt/β-catenin pathway and enhance the osteogenesis-regulated gene expression of ALP, OCN and Col-I. Furthermore, the LaPO_4_/CS scaffolds enhanced bone regeneration and collagen fibre deposition in rat critical-sized calvarial defect sites. In summary, the novel LaPO_4_/CS scaffolds provide an admirable and promising platform for the treatment of bone defects.

## Additional file

**Additional file 1: Figure S1.** The hydrodynamic size distribution of LaPO_4_ agglomerates. **Figure S2.** TG–DTA of samples: (a) LaPO4/CS scaffolds; (b) β-TCP/CS scaffolds. **Figure S3.** XRD patterns of samples: (a) CS powders; (b) β-TCP particles; (c) β-TCP/CS scaffolds. **Figure S4.** FTIR spectra of samples: (a) CS powders; (b) β-TCP particles; (c) β-TCP/CS scaffolds. **Figure S5.** β-TCP/CS scaffold: (a) FESEM images; (b) EDS spectrum. **Figure S6.** In vitro release profile of La_3_ + ions from LaPO_4_/CS scaffolds. In vitro release tests of LaPO4/CS scaffolds were carried out after 0.05 g of the samples was soaked in 5 mL deionized water. At different time points, the concentrations of La_3_ + ions were analysed via inductively coupled plasma/optical emission spectrometry (ICP; iCAP 7000, Thermo Fisher).

## Abbreviations

LaPO_4_: lanthanum phosphate
CS: chitosan
3D: three dimensional
BMSCs: bone marrow mesenchymal stem cells
Col-I: Collagen I
β-TCP: beta-tricalcium phosphate
HA: hydroxyapatite
BG: bioactive glass
BMP-2: bone morphogenetic protein 2
La: Lanthanum
REEs: rare earth elements
S–D: Sprague–Dawley
RT: room temperature
SEM: scanning electron microscopy
EDS: energy dispersive spectrometry
HRTEM: high resolution transmission electron microscope
SAED: selected area electron diffraction
XRD: X-ray powder diffraction
FTIR: Fourier transform infrared spectroscopy
PBS: phosphate buffer saline
CCK-8: Cell Counting Kit 8
ALP: alkaline phosphatase
pNPP: *p*-nitrophenyl-phosphate
BCIP/NBT: 5-bromo-4-chloro-3-indolyl phosphate/tetranitroblue tetrazolium chloride
ECM: extracellular matrix
mRNA: messenger RNA
M-MLV: Moloney Murine Leukemia Virus
RT-PCR: reverse transcription-polymerase chain reaction
GAPDH: glyceraldehyde-3-phosphate dehydrogenase
p-GSK3β: phosphorylated glycogen synthase kinase-3β
GSK3: glycogen synthase kinase-3β
micro-CT: micro-computerized tomography
BMD: bone mineral density
BV/TV: bone volume/tissue volume
VG: Van Gieson’s
OCN: osteocalcin
