# Supplementary material for: Lanthanum phosphate/chitosan scaffolds enhance cytocompatibility and osteogenic efficiency via the Wnt/β-catenin pathway
Source: J Nanobiotechnology. 2018 Nov 29;16:98. doi: 10.1186/s12951-018-0411-9 (PMC6263548; doi:10.1186/s12951-018-0411-9)
Supplement: Supplementary file 1 — Additional file 1: Figure S1. The hydrodynamic size distribution of LaPO4 agglomerates. Figure S2. TG–DTA of samples: (a) LaPO4/CS scaffolds; (b) β-TCP/CS scaffolds. Figure S3. XRD patterns of samples: (a) CS powders; (b) β-TCP particles; (c) β-TCP/CS scaffolds. Figure S4. FTIR spectra of samples: (a) CS powders; (b) β-TCP particles; (c) β-TCP/CS scaffolds. Figure S5. β-TCP/CS scaffold: (a) FESEM images; (b) EDS spectrum. Figure S6. In vitro release profile of La3 + ions from LaPO4/CS scaffolds. In vitro release tests of LaPO4/CS scaffolds were carried out after 0.05 g of the samples was soaked in 5 mL deionized water. At different time points, the concentrations of La3 + ions were analysed via inductively coupled plasma/optical emission spectrometry (ICP; iCAP 7000, Thermo Fisher). [file 12951_2018_411_MOESM1_ESM.docx]

**Supplementary Materials**

**
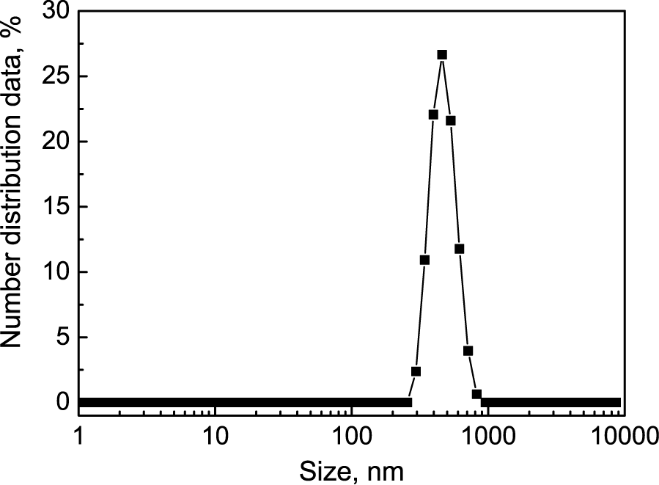
**

Figure S1 The hydrodynamic size distribution of LaPO_4_ agglomerates.

**
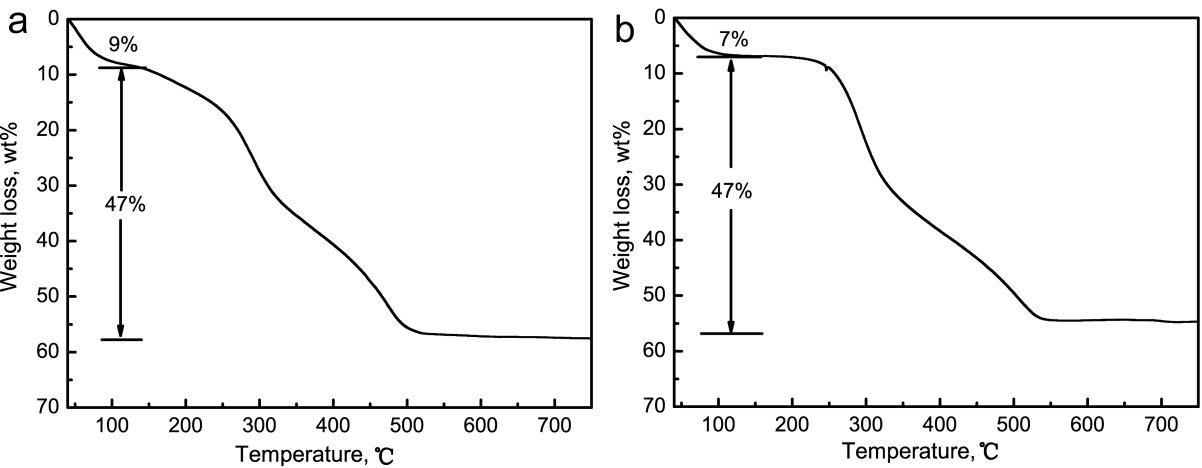
**

Figure S2 TG-DTA of samples: (a) LaPO_4_/CS scaffolds; (b) β-TCP/CS scaffolds.


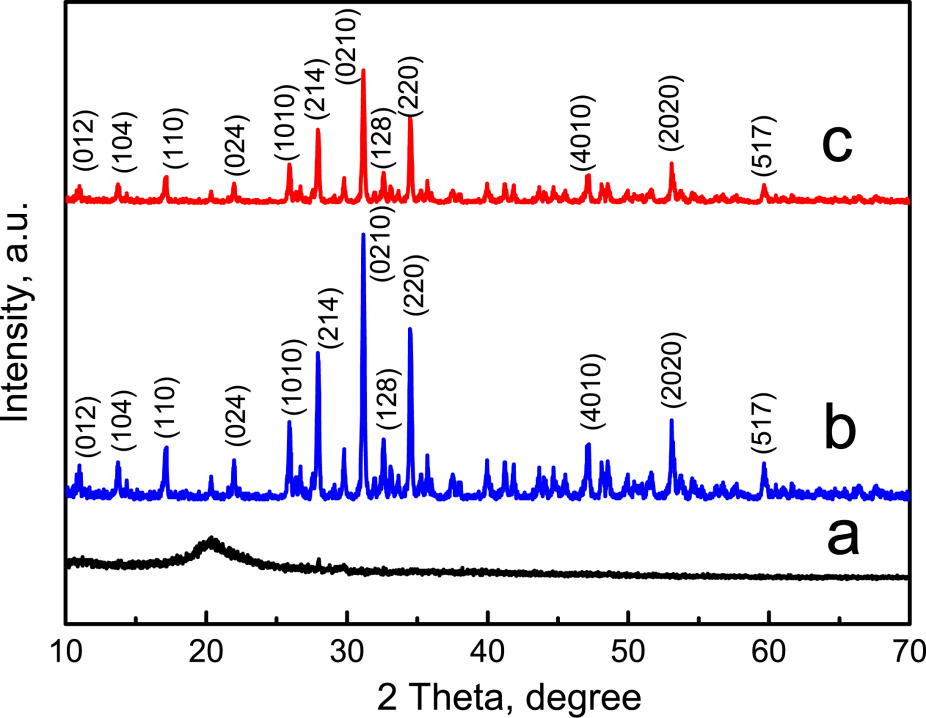


Figure S3. XRD patterns of samples: (a) CS powders; (b) β-TCP particles; (c) β-TCP /CS

scaffolds.


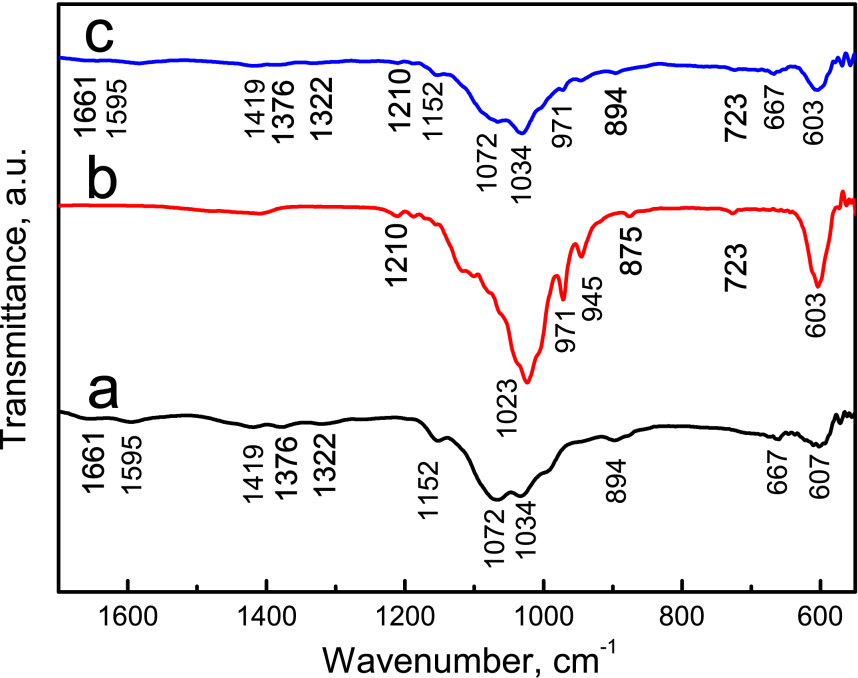


Figure S4. FTIR spectra of samples: (a) CS powders; (b) β-TCP particles; (c) β-TCP/CS scaffolds.


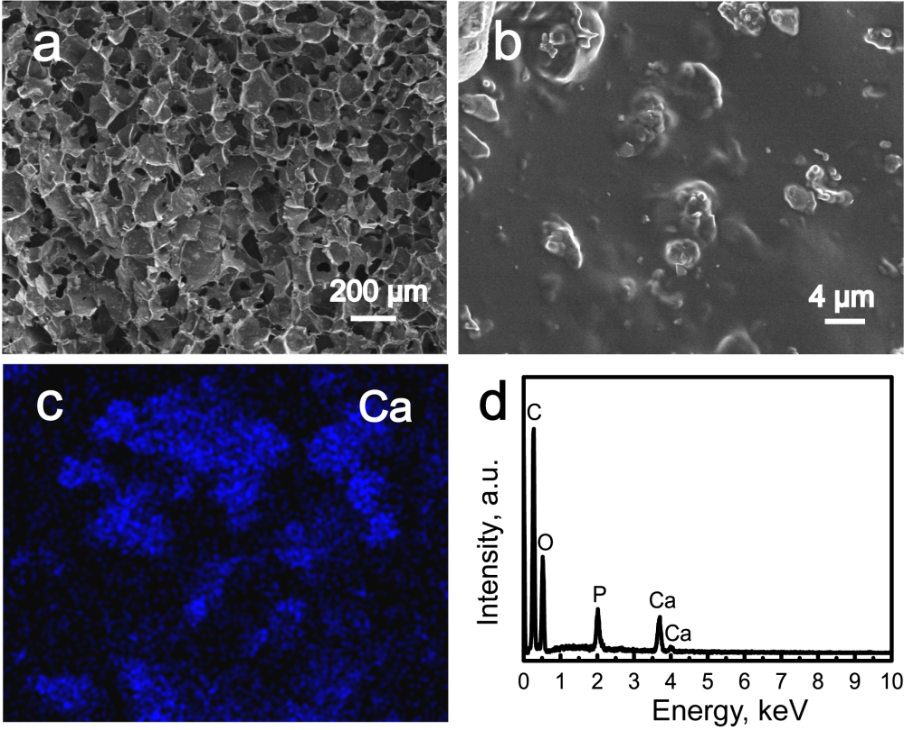


Figure S5. β-TCP/CS scaffold: (a) FESEM images; (b) EDS spectrum.


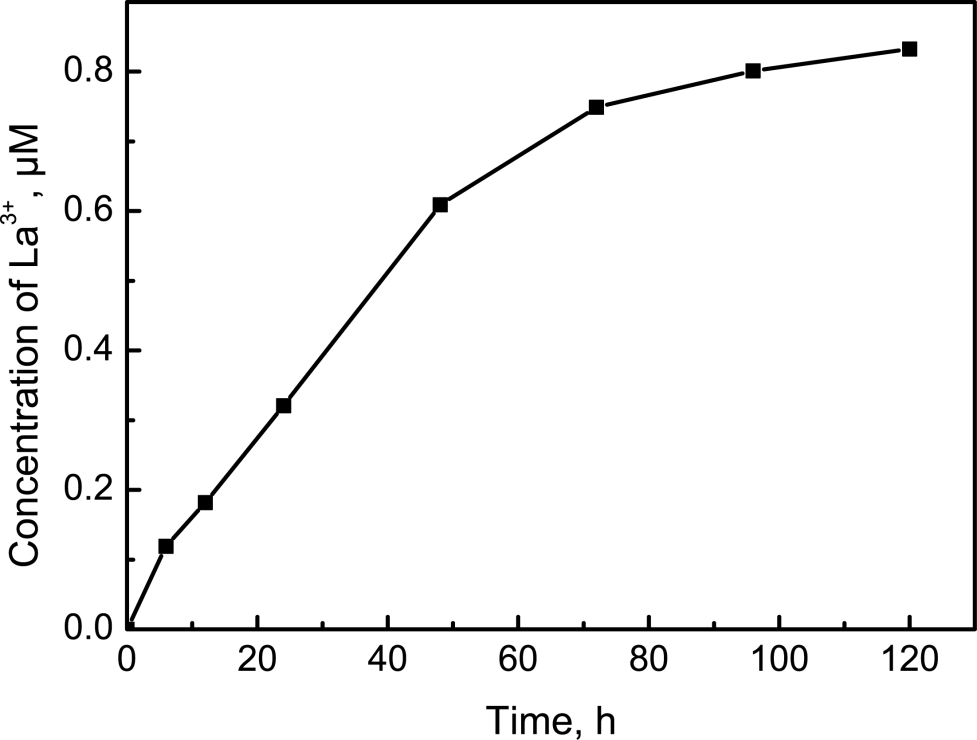


Figure S6. In vitro release profile of La^3+^ ions from LaPO_4_/CS scaffolds. The In vitro release

tests of LaPO_4_/CS scaffolds were carried out after 0.05 g samples were soaked in 5 ml deionized

water. At the different time points, the concentrations of La^3+^ ions were analyzed by inductively

coupled plasma/optical emission spectrometry (ICP; iCAP 7000, Thermo Fisher).
